# Maximizing Leaves, Inflorescences, and Chemical Composition Production of *Moringa oleifera* Trees under Calcareous Soil Conditions

**DOI:** 10.3390/plants11020234

**Published:** 2022-01-17

**Authors:** Amira K. G. Atteya, Aishah N. Albalawi, Hala M. Bayomy, Eman S. Alamri, Esmail A. E. Genaidy

**Affiliations:** 1 Horticulture Department, Faculty of Agriculture, Damanhour University, Damanhour 22516, Egypt; 2 Department of Analytical Chemistry, University College of Haql, Tabuk University, Tabuk 71491, Saudi Arabia; An.albalawi@ut.edu.sa; 3 Department of Nutrition and Food Science, Tabuk University, Tabuk 71491, Saudi Arabia; hala.biomy@agr.dmu.edu.eg (H.M.B.); ialamri@ut.edu.sa (E.S.A.); 4 Department of Food Science and Technology, Damanhour University, Damanhour 22516, Egypt; 5 Pomology Department, Agricultural and Biology Research Institute, National Research Centre, Giza 12622, Egypt; esmail_nrc@yahoo.com

**Keywords:** *Moringa oleifera* tree, growth, leaf yield, inflorescence yield, total phenolic, flavonoid content, antioxidant activity, calcareous soil, vermicompost, nano-fertilization

## Abstract

One of the main issues limiting plant productivity is a lack of soil nutrient resources, especially in calcareous soil, which covers more than one third of the world’s land surface area. On the other hand, despite rising demand for all parts of the *Moringa oleifera* tree, several studies have focused on its leaf production as an herbaceous plant, rather than as a tree, and no extensive research has been carried out on leaf and inflorescence production in the mature tree. As a result, the influence of vermicompost and NPK (minerals and nanoparticles), as well as their combination, under calcareous soil conditions, was investigated in this study. The experiment was set up as a split plot in Randomized Complete Block Design (RCBD) with three replicates. In both seasons considered in this study, it was discovered that increasing the quantity of vermicompost and employing NPK fertilization, as well as their combination treatments, greatly enhanced all parameters and yield of distinct *Moringa oleifera* tree parts. Furthermore, the combination treatment T21 yielded the highest mean values of growth, leaves, and chemical composition parameters, as well as the highest yield from the *Moringa oleifera* tree. In both seasons, the highest number of inflorescences per tree, as well as the yield of fresh and dry inflorescences, was measured using combination treatment T18. In both seasons, however, increasing the level of vermicompost, NPK, and their combination treatments significantly reduced the total phenolic and flavonoid content and antioxidant activity of dry *Moringa oleifera* leaves.

## 1. Introduction

The main factor in agriculture is soil, which provides the nutrient content and affects the strength of plants. Agricultural production in calcareous soils faces numerous challenges due to high infiltration rates, low level of organic matter, water holding capacity, cation-exchange capacity, clay content, poor structure, loss of nutrients due to leaching or deep percolation, high pH with low availability of nutrients, and a nutritional imbalance between elements. These challenges can make it difficult to achieve the desired plant development and output [1,2,3,4]. Potassium (K) plays an important part in a variety of biochemical and physiological processes that affect plant growth, productivity, and disease resistance. It has the ability to improve photosynthesis, control osmotic conditions, and stimulate carbohydrate metabolism. Plants, therefore, require a lot of K for economic crop production and adaptive responses to the environment [5]. Furthermore, nitrogen (N) is an important ingredient in plants, as it provides the necessary protein, amino acid, and carbohydrate content for optimum growth. The rates of N transformations are increased in alkaline calcareous soils, and the efficiency of N use by plants can be modified. As a result, efficient N management through fertilization in calcareous soils requires minimizing ammonia volatilization and leaching of N [5,6]. Phosphorus is a vital macronutrient that can boost metabolism, root growth, plant development, blooming, and yield [7,8]. Furthermore, both supplemental and native phosphorus availability is decreased under alkaline pH conditions. Its anions generate low-solubility compounds with some other elements, such as calcium and magnesium [9].

Finding a means to improve soil chemical and physical qualities, as well as an effective strategy to provide developing plants with the fertilizers they require without losses, is critical for rapid vegetative growth. Foliar fertilization becomes successive in tree fertilization programs under these conditions, especially with the use of nanofertilizers (i.e., those with a nanoscale of 1 nm–100 nm), which allows them to reach the tissues of plants. Nano-NPK fertilizers through foliar application can decrease or eliminate nutrient interactions with water, micro-organisms, and calcareous soil, as well as lower recommended nutrient levels, thereby decreasing soil pollution [10]. Many studies focused on peanut [11], soybean [12], eggplant [13], and olive [14] have stressed the advantages of nanofertilizers for crop cultures. 

Vermicompost as a nonchemical source of plant nutrition is made by earthworms vermicomposting organic material. Earthworms may devour an enormous spectrum of natural leftovers, the result of which has beneficial chemical and physical effects for both plant growth and soil structure [15]. This is due to moisture retention, aggregation formation and resistance, compressibility, and thermal properties, as well as the ability to stimulate and improve nutrient uptake and provide biological control for plant diseases [16]. Vermicompost can help plants to grow better in calcareous soil in four ways: The first is to boost plant nutrient availability during the mineralization of organic matter. Vermicompost contains a larger group of soil-friendly fungi, bacteria, and actinomycetes [17,18,19,20,21,22]. Organic fertilizer enriches soil with macro and microelements and organic materials, according to [23]. Interactions with soil carbonates and pH effects in calcareous soils reduce soil availability of Mn, Fe, Cu, B, and Zn [24,25]. The second method is to stimulate the production of growth-promoting hormones, such as auxins, gibberellins, and cytokinins, in vermicompost microorganisms [26,27]. As a result, plant development can be improved as well as biotic and abiotic stress tolerance [28,29,30]. The vermicompost facilitates the biological control of plant and soil diseases. The fourth method involves boosting the organic matter content of the soil. In general, biological processes, nutrient cycling, soil structure, and retained soil water of calcareous soil are improved, as well as the activation of H^+^-ATPase in root plasma membranes, when adding vermicompost as an organic fertilizer [31,32,33,34]

Furthermore, the nutrients in vermicompost fertilizer have a slow rate of degradation, which suits trees as perennial plants cultivated in calcareous soil. Furthermore, organic fertilizers may be appropriate for achieving sustainable agriculture goals. Studies have focused on petunias [35], tomatoes [36], chamomile [37], and saffron [38,39] to investigate the growth of plants supplemented with vermicompost. They found that growth, flowering, yield, and some secondary products in medicinal and aromatic plants increased with the used amount of vermicompost. Furthermore, organic fertilizer improves various soil qualities [40]. 

Moringa is a small to medium-sized tree that grows to be about 10 m tall, and is known for its horseradish-like roots, drumstick seedspods, and leaves. It is a member of the Moringaceae family, which has only one genus, Moringa, with 10 to 14 species. *Moringa oleifera* is the most well-known species and is native to Northwest India and widely planted in the tropical and subtropical countries [41,42]. It has also been planted in small areas in Egypt. Moringa is a nutrient-dense plant with a high concentration of vitamins and minerals [43]. Moringa is used for animal feed, cleaning agents, growing alleyways, medicine, biogas, green manure, and other applications. Furthermore, all parts of the Moringa tree can be used to treat a variety of ailments, including high blood pressure, lung disease, and skin infections [44]. Moringa should be grown in soils that are slightly acidic to neutral, well drained, and free of clay [45]. Furthermore, proper fertilization promotes the rapid growth of the Moringa tree and improves its potential to produce a healthy plant [46] with a high production of leaves and inflorescences. Sánchez et al. [47], Isaiah [48], and Dania et al. [49] among others have conducted studies on Moringa trees as an herbaceous plant, focusing on its leaf production under normal conditions. On the other hand, there has not been exhaustive research on the production of leaves, inflorescences, and the chemical composition of leaves in mature Moringa trees. 

The objectives of this study were to study the response of leaves, inflorescences, and chemical composition production of mature Moringa trees to variable rates of vermicompost and NPK (mineral and nanoparticles) fertilizers under calcareous soil conditions.

## 2. Results

### 2.1. Growth Characteristics

**Figure 1:**
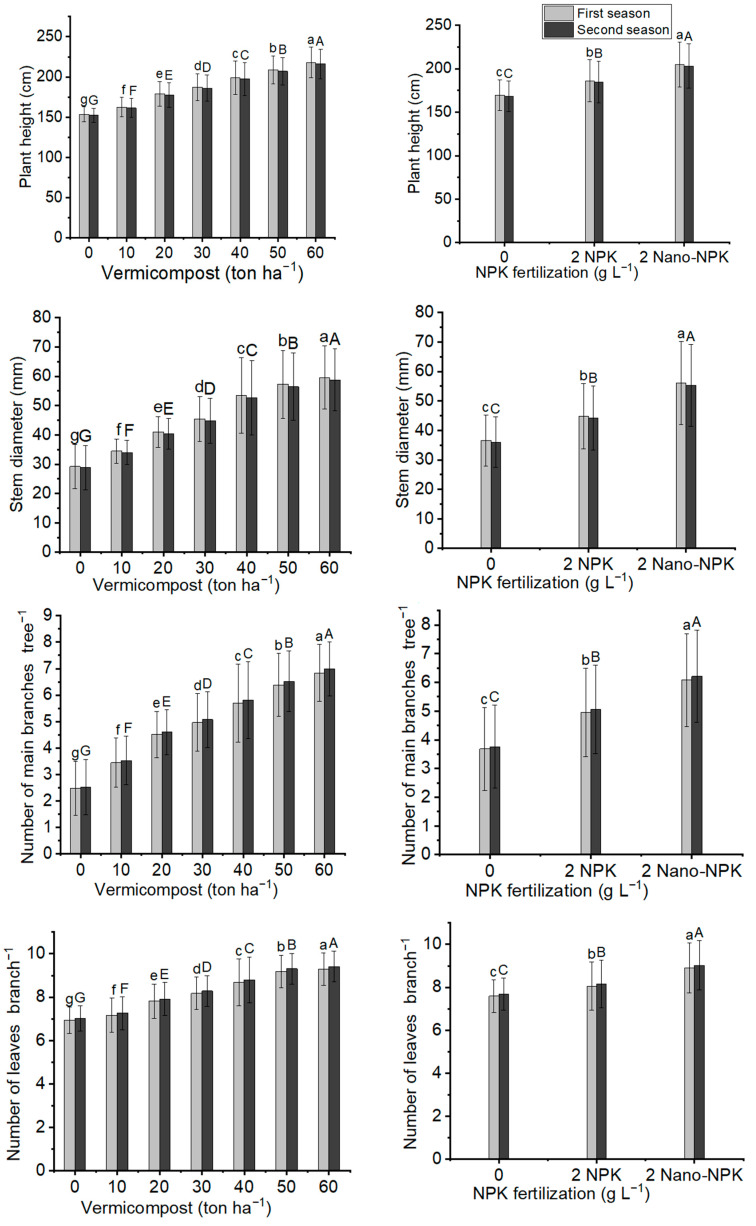
In both seasons of the study, the mean values of plant height (cm), stem diameter (mm), number of main branches tree, and number of leaves per branch of *Moringa oleifera* trees as influenced by vermicompost and NPK fertilization are provided below. The data are presented as a mean with standard error (*n* = 3). Bars with identical lowercase letters are not significant at the 0.05 level of probability.

The growth characteristics of the *Moringa oleifera* tree were significantly affected by the application of organic and NPK fertilization as well as their combination treatments in both seasons. Figure 1 indicates that the addition of 60 ton ha^−1^ of vermicompost led to the best growth characteristics of *Moringa oleifera* trees including significant maximum mean values of plant height (217.8 cm and 216.0 cm), stem diameter (59.60 mm and 58.75 mm), number of main branches per tree (6.84 and 6.99), and number of leaves per branch (9.29 and 9.41), compared to the other vermicompost treatments and control, in the first and second seasons, respectively. Regarding NPK application, using 2 g L^−1^ of Nano-NPK gave the highest significant response to plant height (204.8 cm and 203.1 cm), stem diameter (56.02 mm and 55.22 mm), number of main branches per tree (6.08 and 6.21), and number of leaves per branch (8.90 and 9.02) during both studied seasons, respectively (Figure 1). In addition, for combination treatments, it was observed that the significant tallest plants (240.8 cm and 238.8 cm), maximum mean values of stem diameter (72.57 mm and 71.54 mm), number of main branches per tree (7.99 and 8.17), and number of leaves per branch (10.10 and 10.23) were observed under treatment T21 in both seasons, respectively (Table 1 and Table 2).

### 2.2. Weight and Yield of Fresh and Dry Leaves

**Figure 2:**
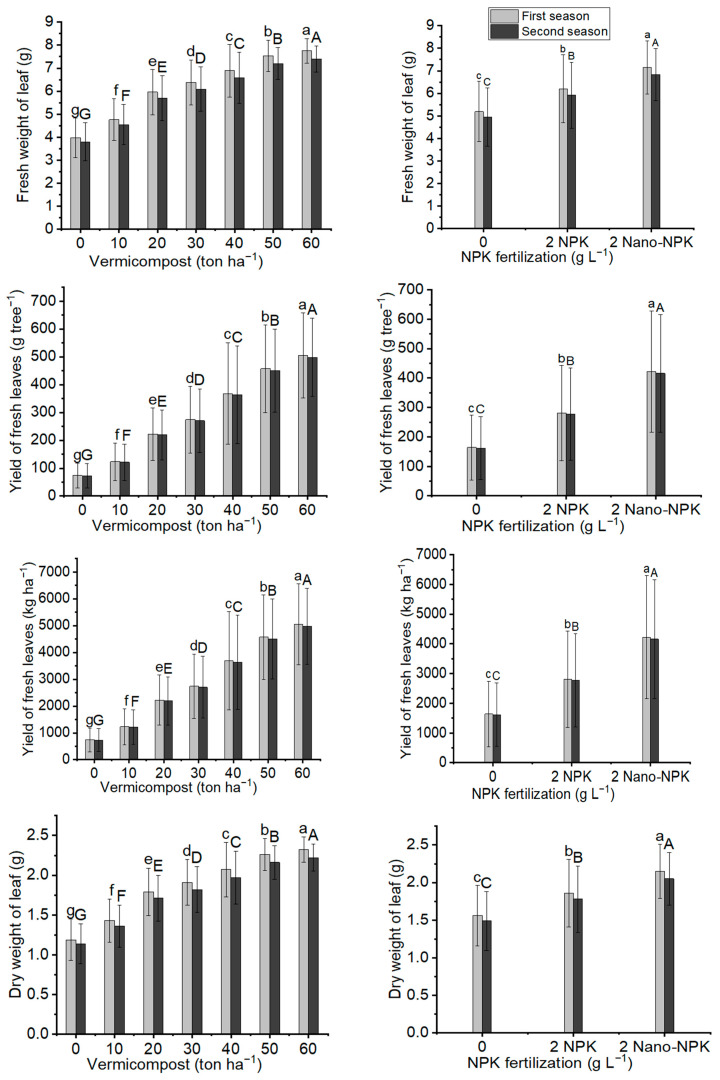
In both seasons of the study, the mean values of fresh weight of leaves (g), yield of fresh leaves (g tree^−1^), yield of fresh leaves (kg ha^−1^), and dry weight of leaf (g) of *Moringa oleifera* trees as influenced by vermicompost and NPK fertilization are provided below. The data are presented as a mean with standard error (*n* = 3). Bars with identical lowercase letters are not significant at the 0.05 level of probability.

**Figure 3:**
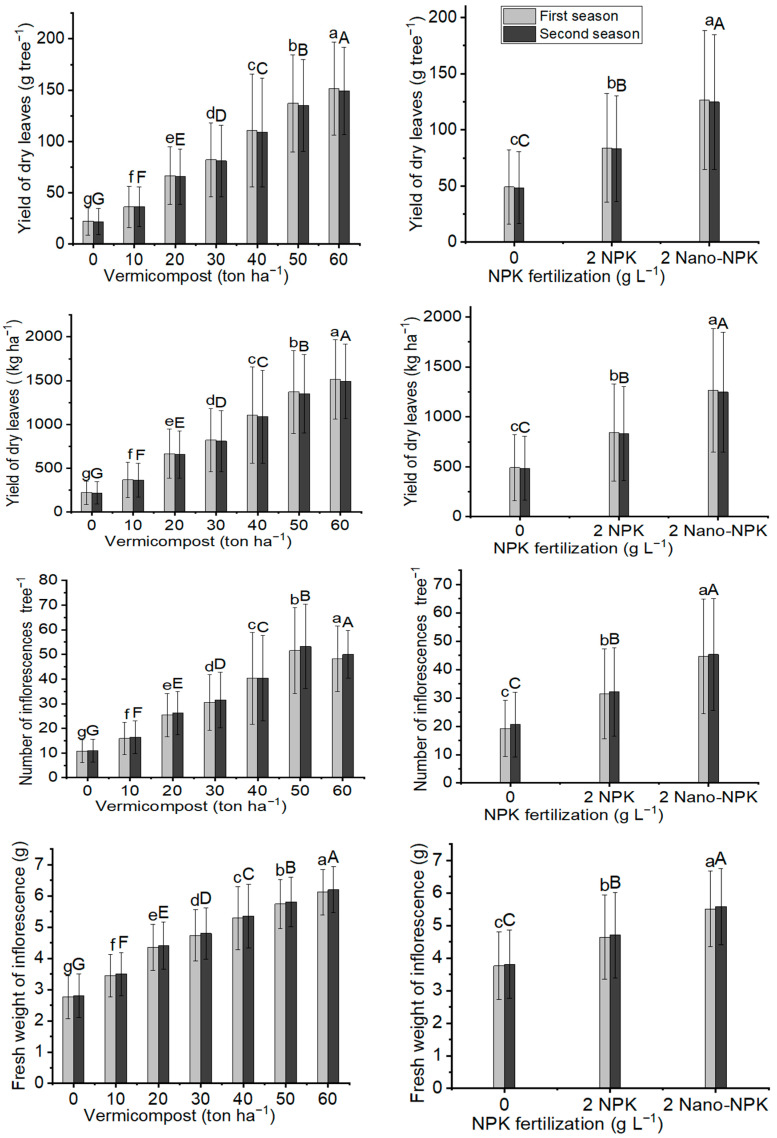
In both seasons of the study, the mean values of yield of dry leaves (g tree^−1^), yield of dry leaves (kg ha^−1^), number of inflorescences per tree, and fresh weight of inflorescence (g) of *Moringa oleifera* trees as influenced by vermicompost and NPK fertilization are provided below. The data are presented as a mean with standard error (*n* = 3). Bars with identical lowercase letters are not significant at the 0.05 level of probability.

The fresh and dry weights of leaves as well as their yield per tree and per hectare were significantly affected by the application of organic fertilization and mineral fertilization, as well as their combination treatments, in both seasons of this study in comparison to the control; see Figure 2 and Figure 3; Table 3 and Table 4. The significant highest mean values of fresh leaf weight (7.75 g and 7.40 g), yield of fresh leaves per tree (505 g tree and 498 g tree^−1^), yield of fresh leaves per hectare (5049 kg ha^−1^ and 4976 kg ha^−1^), dry leaf weight (2.32 g and 2.22 g), yield of dry leaves per tree (151.5 g tree^−1^ and 149.3 g tree^−1^), and yield of dry leaves per hectare (1515 kg ha^−1^ and 1493 kg ha^−1^) in the first and second seasons, respectively, were noticed with the addition of 60 ton ha^−1^ of vermicompost. Moreover, for the mineral fertilization applications, the application of 2 g L^−1^ Nano-NPK had the maximum significant mean values of fresh leaf weight (7.15 g and 6.83 g), yield of fresh leaves per tree (422 g tree^−1^ and 416 g tree^−1^), yield of fresh leaves per hectare (4221 kg ha^−1^ and 4159 kg ha^−1^), dry leaf weight (2.15 g and 2.05 g), yield of dry leaves per tree (126.6 g tree^−1^ and 124.8 g tree^−1^), and yield of dry leaves per hectare (1266 kg ha^−1^ and 1248 kg ha^−1^) in both seasons, respectively. When comparing combination treatments, the maximum mean values of fresh leaf weight (8.33 g and 7.95 g), yield of fresh leaves per tree (674 g tree^−1^ and 664 g tree^−1^), yield of fresh leaves per hectare (6739 kg ha^−1^ and 6641 kg ha^−1^), dry leaf weight (2.50 g and 2.39 g), yield of dry leaves per tree (202.2 g tree^−1^ and 199.2 g tree^−1^), and yield of dry leaves per hectare (2022 kg ha^−1^ and 1992 kg ha^−1^) in both seasons, respectively (Table 3, Table 4 and Table 5) were observed with the application of treatment T21.

### 2.3. Inflorescences Parameters

**Figure 4:**
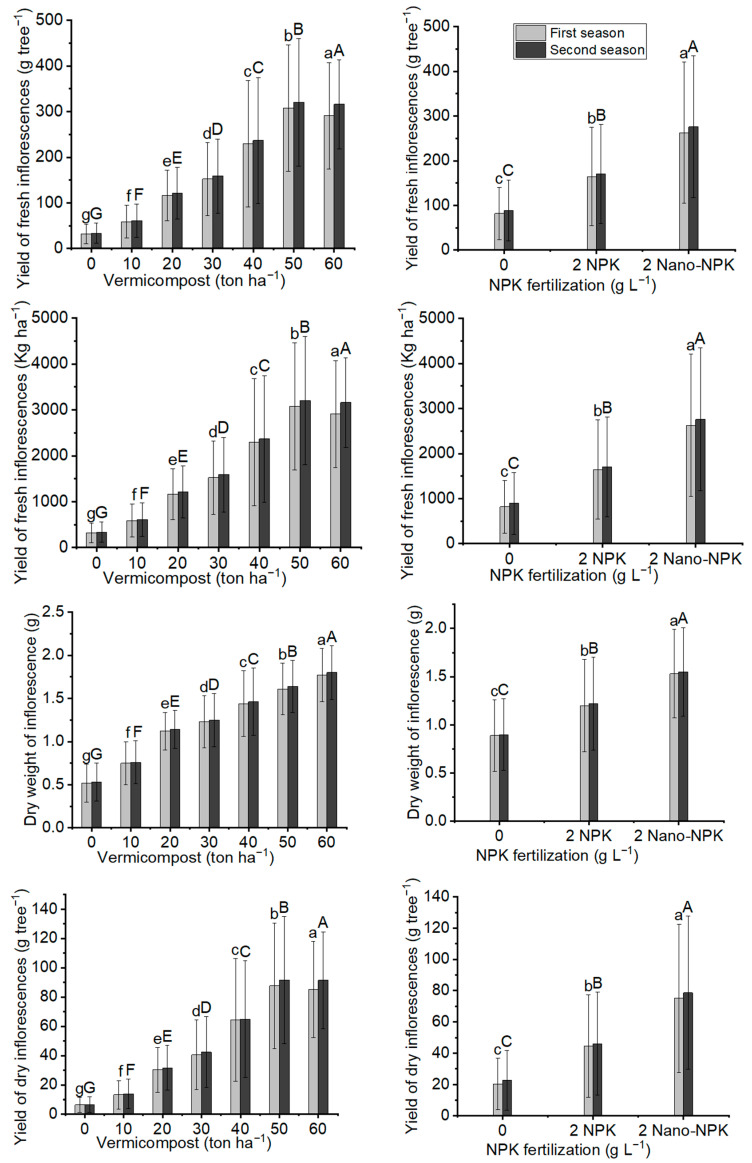
In both seasons of the study, the mean values of yield of fresh inflorescences (g tree^−1^), yield of fresh inflorescences (kg ha^−1^), and dry weight of inflorescence (g), and yield of dry inflorescences (g tree^−1^) of *Moringa oleifera* trees as influenced by vermicompost and NPK fertilization are provided below. The data are presented as a mean with standard error (*n* = 3). Bars with identical lowercase letters are not significant at the 0.05 level of probability.

All NPK and vermicompost levels, as well as their combinations treatments, significantly affected inflorescence parameters of the *Moringa oleifera,* as well as their yield, at the 0.05% probability level. According to the results in Figure 3 and Figure 4; Table 4 and Table 5, it was observed that the maximum mean values of the fresh weight of inflorescences (6.12 g and 6.20 g) and the dry weight of inflorescences (1.77 g and 1.80 g) were recorded with the dose of 60 ton ha^−1^ vermicompost in the first and second seasons, respectively. Meanwhile, the highest mean values of inflorescence numbers per tree (51.6 and 53.2) were noticed with the dose of 50 ton ha^−1^ vermicompost in two seasons, respectively. With NPK fertilizer, 2 g L^−1^ of Nano-NPK significantly gave the highest response to the number of inflorescences per tree (44.7 and 45.3) and the fresh weight of inflorescences (5.51 and 5.58) between the NPK and vermicompost combination treatments. Moreover, the maximum mean values of the fresh weight of inflorescences (6.95 g and 7.04 g) and the dry weight of inflorescences (2.13 g and 2.16 g) were found with combination treatment T21 in both seasons, respectively. On the other hand, the highest mean values of inflorescences number per tree (70.6 and 73.5) in the two studied seasons, respectively, were found with the plants provided treatment T18 in both seasons, respectively.

### 2.4. Yield of Inflorescences 

**Figure 5:**
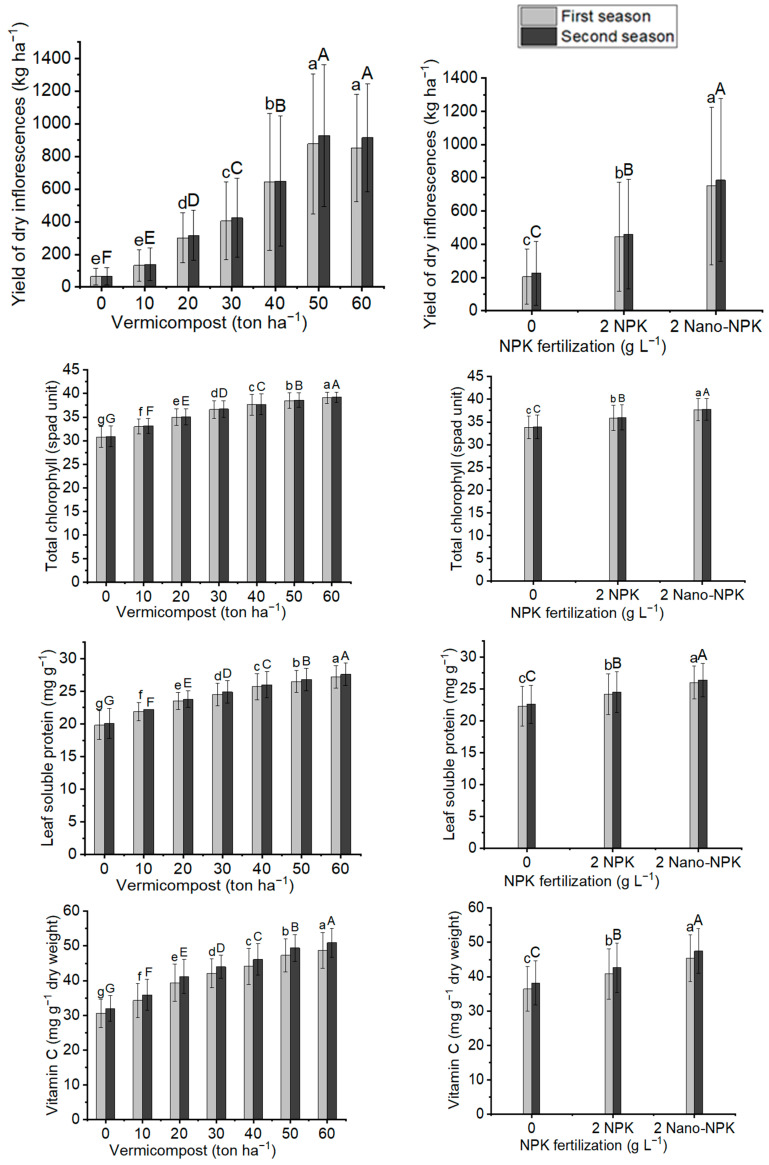
In both seasons of the study, the mean values of yield of dry inflorescences (kg ha^−1^), total chlorophyll (spad unit), leaf soluble protein (mg g^−1^), and vitamin c content (mg g^−1^ dry weight) of *Moringa oleifera* trees as influenced by vermicompost and NPK fertilization are provided below. The data are presented as a mean with standard error (*n* = 3). Bars with identical lowercase letters are not significant at the 0.05 level of probability.

Figure 4 and Figure 5, as well as Table 5 and Table 6, illustrate that all NPK and vermicompost levels, as well as their combination treatments, significantly affected the fresh and dry inflorescence yields of *Moringa oleifera* at the 0.05% probability level. According to the results in Table 7, Table 8 and Table 9, with a 50 ton ha^−1^ vermicompost dose, the maximum mean values of fresh inflorescence yield per tree (307.8 g tree^−1^ and 320.6 g tree^−1^) and per hectare (3078 kg ha^−1^ and 3206 kg ha^−1^), and yield of dry inflorescences per tree (87.7 g tree^−1^ and 91.6 g tree^−1^) and per hectare (877 kg ha^−1^ and 927 kg ha^−1^) were observed in the two seasons, respectively. In terms of NPK fertilizer treatment, using 2 g L^−1^ of Nano-NPK resulted in the maximum response output of fresh inflorescences per tree (262.7 g tree^−1^ and 276.0 g tree^−1^) and per hectare (2627 kg ha^−1^ and 2760 kg ha^−1^), and yield of dry inflorescences per tree (75.2 g tree^−1^ and 78.6 g tree^−1^) and per hectare (752 kg ha^−1^ and 786 kg ha^−1^) in the two seasons, respectively. The interaction between the NPK and vermicompost treatments was substantial in the combination treatments. Furthermore, the plants provided treatment T18 had the highest mean values of fresh inflorescences per tree (461.8 g tree^−1^ and 485.9 g tree^−1^) and per hectare (4618 kg ha^−1^ and 4859 kg ha^−1^) in the first and second seasons, respectively, as well as yield of dry inflorescences per tree (135.8 g tree^−1^ and 143.2 g tree^−1^) and per hectare (1358 and 1432).

### 2.5. Total Chlorophyll, Leaf Soluble Protein, and Vitamin C Contents

It is clear from Figure 5 and Table 6 and Table 7 that the studied treatments of vermicompost, NPK, and their combination treatments in both seasons led to significant differences for total chlorophyll, leaf soluble protein, and vitamin C of the *Moringa oleifera* leaves in both seasons. They increased with increasing vermicompost level. Moreover, this increase reached its maximum level with the application of 60 ton ha^−1^ vermicompost for total chlorophyll (39.1 spad unit and 39.2 spad unit), leaf soluble protein (27.2 mg g^−1^ and 27.6 mg g^−1^), and vitamin C (48.7 mg g^−1^ and 50.9 mg g^−1^) in the two seasons, respectively, compared with the other levels of vermicompost. Meanwhile, the application of 2 g L^−1^ Nano-NPK led to the maximum mean values of total chlorophyll (37.7 spad unit and 37.8 spad unit), leaf soluble protein (26.0 mg g^−1^ and 26.4 mg g^−1^) and vitamin C (45.4 mg g^−1^ and 47.5 mg g^−1^), compared with the other NPK treatments in the two seasons, respectively. In a comparison of combination treatments, the maximum mean values of total chlorophyll (40.3 spad unit and 40.4 spad unit), leaf soluble protein (28.9 mg g^−1^ and 29.3 mg g^−1^), and vitamin C content (53.7 mg g^−1^ and 56.1 mg g^−1^) were noticed with the treatment T21 in the first and second seasons, respectively.

### 2.6. Total Phenoliccontent, Flavonoid Content and Antioxidant Activity

**Figure 6:**
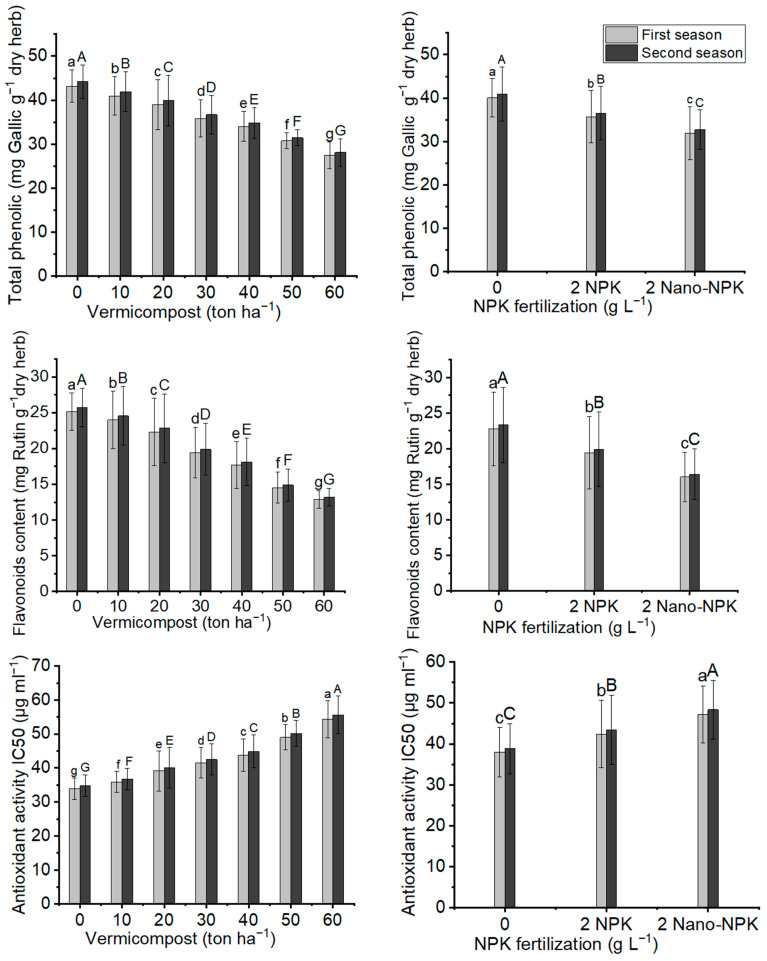
In both seasons of the study, the mean values of total phenolic (mg Gallic g ^−1^ dry herb), flavonoids content (mg Rutin g^−1^ dry herb), and antioxidant activity IC_50_ (µg mL^−1^) of *Moringa oleifera* trees as influenced by vermicompost and NPK fertilization are provided below. The data are presented as a mean with standard error (*n* = 3). Bars with identical lowercase letters are not significant at the 0.05 level of probability.

From Figure 6 and Table 8 it is obvious that in contrast to the other studied parameters in this experiment, the total phenolic content, flavonoid content, and antioxidant activity of *Moringa oleifera* dry leaves decreased significantly after the application of vermicompost, NPK, and their combination treatments in both seasons. This means that the maximum mean values of total phenolic content (43.16 mg Gallic g^−1^ dry herb and 44.17 mg Gallic g^−1^ dry herb), flavonoid content (25.13 mg Rutin g^−1^ dry herb and 25.72 mg Rutin g^−1^ dry herb), and antioxidant activity (33.98 µg ml^−1^ for 50% inhibition and 34.77 µg ml^−1^ for 50% inhibition) were found with 0 ton ha^−1^ vermicompost (control) in the two seasons, respectively, compared with all levels of vermicompost. Furthermore, the application of 0 g L^−1^ NPK (control) gave the maximum mean values of total phenolic content (40.02 g Gallic g^−1^ dry herb and 40.96 g Gallic g^−1^ dry herb), flavonoid content (22.80 mg Rutin g^−1^ dry herb and 23.34 mg Rutin g^−1^ dry herb), and antioxidant activity (37.95 µg mL^−1^ for 50% inhibition and 38.84 µg mL^−1^ for 50% inhibition) compared with both treatments of NPK in the two seasons, respectively. For combination treatments, the maximum mean values of total phenolic content (47.12 g Gallic g^−1^ dry herb and 48.23 g Gallic g^−1^ dry herb), flavonoid content (28.01 mg Rutin g^−1^ dry herb and 28.67 mg Rutin g^−1^ dry herb), and antioxidant activity (30.70 µg mL^−1^ for 50% inhibition and 31.42 µg mL^−1^ for 50% inhibition) were recorded with treatment T1 (control treatment) in the two seasons, respectively.

### 2.7. Phosphorus and Potassium Contents 

**Figure 7:**
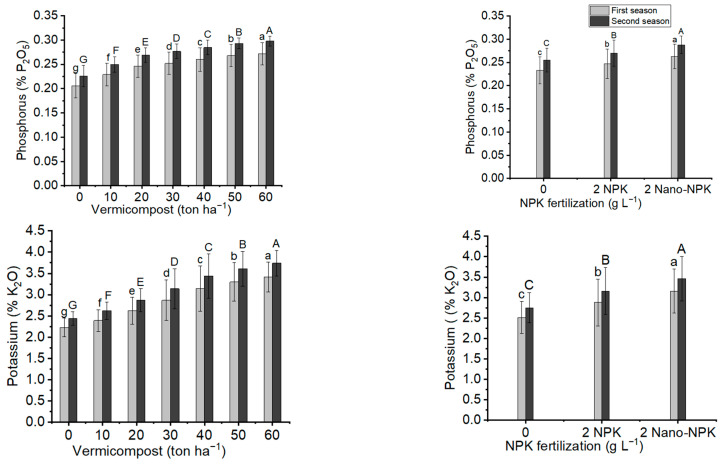
In both seasons of the study, the mean values of phosphorus content (% P_2_O_5_) and potassium content (% K_2_O) of *Moringa oleifera* trees as influenced by vermicompost and NPK fertilization are provided below. The data are presented as a mean with standard error (*n* = 3). Bars with identical lowercase letters are not significant at the 0.05 level of probability.

The phosphorus and potassium content results of *Moringa oleifera* leaves provided in Figure 7 and Table 9 show that the phosphorus and potassium contents increased significantly with the application of vermicompost, NPK, and their combination treatments in both seasons. In comparison to the other levels of vermicompost, the maximum mean values of phosphorus content (0.272% P_2_O_5_ and 0.298% P_2_O_5_) and potassium content (3.41% K_2_O and 3.74% K_2_O) were observed with the application of 60 ton ha^−1^ vermicompost in the two seasons, respectively. In addition, when compared to the other NPK treatments, the administration of 2 g L^−1^ Nano-NPK resulted in the highest mean values of phosphorus content (0.263% P_2_O_5_ and 0.288% P_2_O_5_) and potassium content (3.16% K_2_O and 3.46% K_2_O) in both seasons. In the first and second seasons, the treatment T21 resulted in the highest mean values of phosphorus (0.282% P_2_O_5_ and 0.309% P_2_O_5_) and potassium contents (3.63% K_2_O and 3.98% K_2_O), respectively.

## 3. Discussion

### 3.1. The Impact of Calcareous Soil on Moringa oleifera Growth Characteristics, Yield, and Chemical Constituents

The seeds of *Moringa oleifera* were able to emerge in calcareous soil without any organic or inorganic fertilization treatments in this experiment (control treatment). The seedlings, on the other hand, grew slowly after that. Finally, they created a miniature tree that resembled a narrow branch with a modest number of leaves and inflorescences. These showed the minimal mean values of growth parameters, yield of fresh and dried leaves and inflorescences, as well as the content of chemical substances including vitamin C, potassium, and phosphorus in the *Moringa oleifera* leaves. In contrast, in the extract from the *Moringa oleifera* dry leaves of the control trees, the highest mean values of total phenolic content, flavonoid content, and antioxidant activity were observed. In calcareous soil, this could be due to a lack of soil nutrients and their accessibility. Leaching, deep percolation, and nitrogen transformations all contribute to nitrogen loss, low availability of phosphorous and micronutrients, as well as imbalances among potassium, magnesium, calcium, and other elements [1,2,3,50]. 

These findings are in line with those of Haukioja et al. [51], who discovered that when N is scarce, the metabolism of developed plants shifts toward the synthesis of non-N-containing secondary metabolites, such as phenolic and terpenoids. According to Bavaresco and Poni [52], calcareous soil lowers P and K levels in various organs of the plant, reducing overall canopy photosynthesis. As a result, the plant’s dry matter suffers, resulting in lower inflorescence production as Khan and Qasim [53] found in wheat plants grown in calcareous soil. When compared to supplements with compost and spent grain, squash germination parameters recorded the lowest values after 15 days of seeding in calcareous soil, according to Aboukila et al. [4]. Semida et al. [54] found that untreated plants growing in salty calcareous soil had the lowest growth metrics, total soluble sugar concentrations, free proline, anthocyanin concentrations, and photosynthetic efficiency.

### 3.2. Effects of Vermicompost on Moringa oleifera Tree Yield, Growth Parameters, and Its Chemical Components

The collected results demonstrate that increasing the amount of vermicompost used boosts the improvement in all analyzed parameters throughout the course of the two seasons considered in the study. This improvement could be attributed to the role of endogenous auxins, gibberellins, and cytokinins in regulating growth in vermicompost. Furthermore, mineralizing organic matter, increasing soluble forms of nutrients by modifying soil pH, and promoting element uptake by roots are all ways to increase the availability of plant nutrients in the soil, such as nitrogen, phosphorus, potassium, and microelements [22,55,56]. Furthermore, utilizing vermicompost improves the physical and chemical qualities of calcareous soil. Due to its slow rate of nutrient breakdown, vermicompost fertilizer is suited for *Moringa oleifera* trees, due to their extended growing season [35,57]. Other researchers have found that increased vermicompost consumption improved vegetative features and increased leaf and flower number, length, fresh and dry weight, total chlorophyll, greenness, yield, and plant secondary products in plants [38,39,58]. 

Furthermore, Arancon et al. [35] (in petunias), Atiyeh et al. [59] (in marigolds), Arancon et al. [60] (in strawberries), Liuc and Pank [61] (in Roman Chamomile), and Muscolo et al. [62] (in carrots) all discovered that the application of vermicompost increased growth, blooming parameters, and all quantitative and qualitative parameters. Furthermore, vermicompost has been found to improve the nutritional quality of the plants as well as development, flowering, yield, and chemical composition of pakchoi [56], strawberries [63], lettuce [64], and Chinese cabbage [65]. These findings are consistent with those of [62,63,64,65,66,67,68,69], who observed a considerable increase in yield and accumulation of N, P, K, Ca, and Mg in the root and shoot system after treating plants with vermicompost or its water-extractable fraction. This could be linked to the role of vermicompost in enhancing plant nutrient availability in the soil by adding micro and macronutrients during the decomposition of organic matter, modifying soil pH to increase soluble forms of nutrients, and increasing element uptake by the roots [22,55,56]. In addition, while increasing vermicompost enhanced vitamin C content and total carotenoids, it decreased antioxidant activity as well as total phenolics and flavonoids. This could be due to the metabolism of grown plants moving toward the production of N-compounds and away from non-N-containing secondary metabolites, such phenolics and terpenoids under high-N circumstances [70,71]. Lowering N concentrations, mineral nutrient levels, and enhanced phenolic compound accumulation in plant tissue all boost the antioxidant capability of leaves [56,72,73,74]. Moreover, despite the discovery by Law-Ogbomo et al. [75] that applying poultry manure to okra plants increased growth, yield, and P, K, Na, and Mn content, vermicompost is preferable over manure and plant compost in most situations, according to Ngo et al. [76] and Aryal and Tamrakar [77]. The application of vermicompost resulted in increased height, diameter, and yield, when compared to farmyard manure.

### 3.3. Effects of NPK Fertilizer on Moringa oleifera Growth Parameters, Yield, and Chemical Components

N, P, and K are the three most important nutrients for crops, and a deficiency of any of these elements during crop growth is well known to have a negative impact on the plant’s reproductive ability, growth, and yield [78,79,80]. These elements are in charge of a multitude of enzymatic and metabolic processes, as well as seed, pod, inflorescence, shoot, and root health. In both considered seasons, and under alkaline calcareous soil conditions, foliar application of Nano-NPK surpassed the ground application of NPK in reaching the best mean values of the examined growth traits, yield, and chemical components of *Moringa oleifera* trees, according to the findings of this study. Foliar fertilization has a better chance of resolving nutritional deficits in plants caused by poor nutrient transport to the roots and, in alkaline calcareous soils, it is usually more cost-effective and efficient [81,82,83]. Normal fertilizers, on the other hand, are typically lost to the environment and are incapable of being absorbed by plants, resulting in considerable economic and resource losses as well as serious environmental damage [84]. As they have a nanoscale of 1 nm–100 nm, nanofertilizers have demonstrated excellent results at optimum concentrations, allowing them to penetrate plant leaves, which are the basic units for gas exchange, photosynthesis, and transpiration [85,86] as well as to reduce nutrient requirements and increasing plant productivity [87]. Nanofertilizers can be sprayed on plants to avoid interactions with water, microbes, and soil (e.g., calcareous soil), as well as to boost plant parameters and yields [10,73,88]. Many researchers have concurred with these findings [89,90]. Haukioja et al. [51] found that when nitrogen was abundant, plants predominantly formed molecules with high nitrogen content, and their metabolism shifted away from carbon-containing chemicals, such as phenolics and terpenoids. Fuglier [44] discovered that fertilizing Moringa trees with nitrogen and phosphate promoted root development and leaf canopy growth. According to Liu and Lal [91], manufactured nanofertilizers increased the biomass of *Arachis hypogeae* L. by 15%. Fagbenro [92], Ainika and Amans [93], Ghafariyan et al. [94], Mahmoodzadeh et al. [95], and Farnia and Ghorbani [96] have found that the application of NPK compound fertilizer has a considerable impact on crop growth, chemical composition, and yield metrics. Abdel-Aziz et al. [86] found that direct exposure to nanoparticles resulted in a significant boost in all growth metrics, measured at optimal nanosolution concentrations, in wheat plants. The foliar application of nanofertilizers and conventional NPK fertilizers improved plant growth, biomass, grain, photosynthetic pigments, chemical constituents, protein content, and yield, with nanofertilizer applications [74,87,97,98,99,100]. When compared to control treatments, Khalid and Shedeed [101] found that the foliar application of NPK resulted in the highest values of vegetative growth characteristics, such as plant height, leaf number, branch number, capsule number, herb dry weight, yield, and chemical content parameters (e.g., fixed oil percentage, total carbohydrates, soluble sugars, protein, potassium, and phosphorus content). Hasaneen and Abdel-aziz [102] discovered that applying NPK nanoparticles or nanoengineered CNTs-NPK to the leaves of French bean plants increased their growth metrics. Hagagg et al. [14] discovered that treating olive seedlings with Nano-NPK fertilizers at a concentration of 0.2% resulted in the greatest values for leaf and root metrics. Total chlorophyll, as well as N, P, and K uptake, were improved by applying Nano-NPK fertilizer. Abd El Gayed and Attia [28] discovered that NPK (20:20:20) at 4.5 g pot^−1^ had the greatest positive effect on vegetative growth characteristics, number of inflorescences plant^−1^, chlorophyll content (SPAD), total carbohydrate, leaf N, P, and K percentages in cockscomb. The importance of these NPK fertilizers, according to Mokrani et al. [100], lies in their ability to provide the necessary nutrients for plant growth. Sarwar et al. [103] stated that NPK administration aided Moringa growth by producing plants with significantly higher larger height, leaf count, stem girth, and maximum number of branches than other treatments. Soylu et al. [104], Soleimani [105], Arif et al. [106], and Hamayun [107] discovered that foliar treatment of nitrogen, phosphorus, and potassium in several cereal crops resulted in rapid vegetative development, as well as significant increases in the number of leaves, plant height, thousand-grain weight, and yield. Jubeir and Ahmed [108] discovered that employing a nanofertilizer enhanced the dry matter and chlorophyll content of all leaves. Clearly, these treatments improved the vegetative growth date palm and flowering. Alzreejawi and Al-Juthery [109] discovered that Nano-NPK (12-12-36) spray was significantly superior in terms of leaf chlorophyll content, plant height, stem diameter, biological yield, grain yield, and harvest index. The fixation of phosphatic fertilizers in alkaline soils due to calcareousness is one of the key challenges that can reduce maize and soybean yields, according to Rafiullah et al. [110]. 

### 3.4. Effects of Vermicompost–NPK Combination Treatments on Moringa oleifera Tree Yield Growth Parameters, and Chemical Components

Despite the vital role of foliar NPK administration in the quick uptake and translocation and its beneficial impact on growth and yield, foliar fertilization cannot completely substitute for nutrients from the roots. It can only be utilized to reduce the amount of fertilizer applied to the soil [111]. To address this gap, organic amendments, such as adding vermicompost and foliar spraying of Nano-NPK under calcareous soil conditions, are appropriate techniques that can boost the development, yield, and chemical content of *Moringa oleifera* trees. As a result, the maximum mean values of the two combination treatments T18 and T21 were those for plant height, stem diameter, number of main branches per tree, number of leaves per branch, fresh and dry weight of leaves, yield of fresh leaves per tree and per hectare, yield of dry leaves per tree and per hectare, number of inflorescences per tree, fresh and dry weight of inflorescences, yield of fresh inflorescences per tree and per hectare, yield of dry inflorescences per tree and per hectare, total chlorophyll, leaf soluble protein, vitamin C, phosphorus and potassium contents in combination with minimum mean values of total phenolic content, flavonoid content, and antioxidant activity in both seasons considered in the study. Combining organic and inorganic fertilizers has important consequences for plant growth as well as soil chemical and biological features [112,113]. As a result, employing vermicompost, either alone or in combination with mineral fertilizers has a positive effect on plant development and yield [114]. Our findings were similar to those of Bajracharya et al. [115], Bhattarai and Tomar [116], Thakur and Uphoff [117], Zhao et al. [118], Prativa and Bhattarai [119], and Ghimire et al. [120], who all observed that using vermicompost in combination with NPK produced the best results in terms of plant growth and production.

## 4. Materials and Methods

This study was conducted in an open field of a private farm in El-Amiriya, Alexandria Governorate, Egypt, during the two consecutive seasons of 2018/2019 and 2019/2020, in order to investigate the effect of organic and inorganic fertilization on the growth and chemical composition of *Moringa oleifera* trees.

### 4.1. Plant Material

*Moringa oleifera* seeds were planted in February 2018 and February 2019. Distances between rows and within plants in rows were 1 m, respectively. Pest management and other agricultural measures (e.g., irrigation) were used when necessary and as indicated.

### 4.2. Treatment

During February–March 2018/2019 and 2019/2020, the experiment was set up as a split plot arranged in Randomized Complete Block Design (RCBD) with three replicates. The main plots received organic fertilization in the form of a ground dose of vermicompost, whereas the subplots received mineral and Nano-NPK fertilization of *Moringa oleifera* plants (19:19:19). Vermicompost fertilization was applied to the main plot. NPK fertilization was put in the subplot. All feasible combinations of the two factors evaluated were tested (Table 10). The experiment comprised 21 treatments consisting of a mix of vermicompost ground addition (0, 10, 20, 30, 40, 50, and 60 ton ha^−1^ vermicompost) and spraying NPK fertilization (0, 2 g L^−1^ mineral-NPK and 2 g L^−1^ Nano-NPK). Before ten days from planting, the associated amount of vermicompost was added. The 2 g L^−1^ NPK was used as a ground dose, while the 2 g L^−1^ Nano-NPK was used as foliar application. After two weeks, all plants treated with NPK fertilization received one application per week up until six weeks after planting. This was increased to twice a week, until completion of the trial. In addition, Tween 80 (0.01%) was utilized as a wetting agent. Plants that had not been treated with NPK (NPK control) were sprayed with distilled water and Tween 80. 

#### Nano-NPK Preparation

In a 2 L glass beaker, 400 g of 19:19:19 NPK mineral fertilizer was weighed, then 550 mL of distilled water was poured in and swirled until completely dissolved. The clear solution was then heated to 50 °C, after which 50 g of citric acid was added with vigorous stirring for 15 min. Potassium hydroxide was gradually added until the necessary pH was reached. The clear solution changed to a milky look when potassium hydroxide was added, demonstrating the conversion to nanoparticle size. The used concentration was calculated based on the amount of mineral NPK used in the Nano-NPK process. In the first and second seasons, seeds were planted on 1 February. On one side of the row, seeds (three seeds hill^−1^) were sown. The seedlings were trimmed to one plant per hill after 30 days. The plots were weeded as often as feasible every two weeks. Table 11 and Table 12 provide the physical and chemical parameters of the vermicompost and soil samples, as determined by [121,122].

### 4.3. Data Recorded Each Season

#### 4.3.1. Growth and Flowering Parameters

A sample of five plants was taken at random from each replicate (i.e., fifteen plants from every treatment), in order to measure the following growth parameters: Plant height (cm), stem diameter (mm), number of main branches per tree, number of leaves per branch, fresh and dry weight of leaves (g), yield of fresh leaves (g tree^−1^ and kg ha^−1^), yield of dry leaves (g tree^−1^ and kg ha^−1^), number of inflorescences per tree, fresh and dry weight of inflorescences (g), yield of fresh inflorescences (g tree^−1^ and kg ha^−1^), and yield of dry inflorescences (g tree^−1^ and kg ha^−1^).

#### 4.3.2. Total Chlorophyll

Total chlorophyll content (SPAD unit) was quantified using a SPAD-502 Chlorophyll Meter (Minolta Camera Co., Ramsey, NJ, USA).

#### 4.3.3. Leaf Soluble Protein Content (mg g^−1^)

Soluble protein contents of the extracts were determined using Folin–Ciocalteu reagent, following the method described by [123].

#### 4.3.4. Vitamin C (mg g^−1^ dry weight) 

Vitamin C in leaves was determined according to the method described by [124].

#### 4.3.5. Total Phenolic Content (mg Gallic g^−1^ dry herb) 

Total phenolic content was determined according to the method of [125]. 

#### 4.3.6. Total Flavonoid Content (mg Rutin g^−1^ dry herb)

Total flavonoid content was determined using the colorimetric method of [126]. 

#### 4.3.7. Antioxidant Activity Determinations IC_50_ (µg mL^−1^)

The antioxidant activities of different samples were determined using 2- diphenyl-1-picrylhydrazyl radical (DPPH) [127]. The extract concentration (µg mL^−1^) providing 50% of antioxidant activities (IC_50_) was calculated by plotting the inhibition percentage against extract concentration in a graph. 

#### 4.3.8. Phosphorus Percentage (P_2_O_5_) 

Phosphorus percentage was determined in leaves colorimetrically as reported by [122]. 

#### 4.3.9. Potassium Percentage (K_2_O) 

Potassium percentage was determined in leaves by atomic absorption spectrophotometry following the method was described by [128].

### 4.4. Statistical Analysis

The experiment was set up as a split plot arranged in Randomized Complete Block Design (RCBD) with three replicates for every treatment. Vermicompost fertilization was put in the main plot. NPK fertilization was carried out in the subplot. All data from the tested treatments were subjected to analysis of variance using the SAS software [129]. The LSD test was used to compare the means of the treatments at the 5% probability level. The experiment was performed in the second year using the same methods and approaches as the first year.

## 5. Conclusions

Increasing the amount of vermicompost improved the characteristics of the *Moringa oleifera* tree, as well as the yield of its leaves and inflorescences. Finally, our recommended treatment for maximizing fresh and dry leaf production is treatment T21, while for the highest possible yield of fresh and dry inflorescences, treatment T18 was the best. On the other hand, the control treatment led to the maximum total phenolic content, flavonoid content, and antioxidant activity of *Moringa oleifera* dry leaves.

## Figures and Tables

**Table 1:** The influence of combined treatments of vermicompost and NPK fertilization on the mean values of plant height (cm), stem diameter (mm), and number of main branches per tree of *Moringa oleifera* trees in the first and second seasons.

Vermicompost (ton ha^−1^)	NPK (2 g L^−1^)	Plant Height (cm)		Stem Diameter (mm)		Number of Main Branches Tree^−1^	
		First Season	Second Season	First Season	Second Season	First Season	Second Season
Control	Control	146.6 ± 2.16 u	145.4 ± 2.16 u	20.18 ± 0.20 u	19.89 ± 0.43 s	1.31 ± 0.10 p	1.34 ± 0.02 t
	Mineral	148.7 ± 2.23 t	147.4 ± 2.23 t	29.76 ± 0.30 t	29.33 ± 0.66 r	2.47 ± 0.19 o	2.52 ± 0.04 s
	Nano	165.4 ± 2.43 p	164.1 ± 2.43 p	37.68 ± 0.37 p	37.14 ± 0.80 n	3.65 ± 0.28 lm	3.73 ± 0.06 p
10	Control	151.8 ± 2.19 s	150.6 ± 2.19 s	30.99 ± 0.29 s	30.55 ± 0.63 q	2.48 ± 0.19 o	2.52 ± 0.04 s
	Mineral	158.1 ± 2.32 r	156.8 ± 2.32 r	32.43 ± 0.32 r	31.97 ± 0.69 p	3.36 ± 0.26 n	3.43 ± 0.06 r
	Nano	178.0 ± 2.62 m	176.5 ± 2.62 m	39.94 ± 0.39 m	39.37 ± 0.85 k	4.54 ± 0.35 j	4.64 ± 0.08 m
20	Control	160.2 ± 2.36 q	158.9 ± 2.36 q	35.32 ± 0.34 q	34.81 ± 0.75 o	3.45 ± 0.26 mn	3.53 ± 0.06 q
	Mineral	182.2 ± 2.68 l	180.7 ± 2.68 l	40.39 ± 0.39 l	39.82 ± 0.85 k	4.74 ± 0.36 j	4.84 ± 0.08 l
	Nano	194.7 ± 2.86 i	193.1 ± 2.86 i	47.37 ± 0.46 i	46.7 ± 1.00 h	5.33 ± 0.41 h	5.45 ± 0.09 i
30	Control	169.6 ± 2.49 o	168.2 ± 2.49 o	38.32 ± 0.37 p	37.78 ± 0.81 m	3.75 ± 0.29 kl	3.83 ± 0.07 o
	Mineral	185.3 ± 2.72 k	183.8 ± 2.72 k	42.68 ± 0.42 k	42.07 ± 0.90 j	5.03 ± 0.38 i	5.14 ± 0.09 k
	Nano	207.3 ± 3.05 f	205.6 ± 3.05 f	55.38 ± 0.54 f	54.59 ± 1.17 e	6.12 ± 0.47 ef	6.25 ± 0.11 f
40	Control	172.8 ± 2.54 n	171.3 ± 2.54 n	39.15 ± 0.38 n	38.59 ± 0.83 l	3.95 ± 0.30 k	4.03 ± 0.07 n
	Mineral	205.2 ± 3.02 g	203.5 ± 3.02 g	52.45 ± 0.51 g	51.7 ± 1.11 f	5.92 ± 0.45 fg	6.05 ± 0.11 g
	Nano	218.8 ± 3.22 c	217.0 ± 3.22 c	68.82 ± 0.67 c	67.84 ± 1.46 c	7.21 ± 0.55 c	7.36 ± 0.13 c
50	Control	188.5 ± 2.77 i	186.9 ± 2.77 j	43.74 ± 0.43 j	43.12 ± 0.93 i	5.13 ± 0.39 hi	5.24 ± 0.09 j
	Mineral	209.4 ± 3.08 e	207.7 ± 3.08 e	57.63 ± 0.56 e	56.81 ± 1.22 d	6.32 ± 0.48 e	6.45 ± 0.11 e
	Nano	228.2 ± 3.36 b	226.4 ± 3.36 b	70.43 ± 0.69 b	69.43 ± 1.49 b	7.70 ± 0.59 b	7.87 ± 0.14 b
60	Control	197.9 ± 2.91 h	196.2 ± 2.91 h	48.02 ± 0.47 h	47.34 ± 1.02 g	5.72 ± 0.44 g	5.85 ± 0.10 h
	Mineral	214.6 ± 3.16 d	212.9 ± 3.16 d	58.22 ± 0.57 d	57.39 ± 1.23 d	6.81 ± 0.52 d	6.96 ± 0.12 d
	Nano	240.8 ± 3.54 a	238.8 ± 3.54 a	72.57 ± 0.71 a	71.54 ± 1.54 a	7.99 ± 0.61 a	8.17 ± 0.14 a

At the 0.05 significance level, the means of columns separated by the same lowercase letter do not differ statistically. The data are mean values with standard error (*n* = 3).

**Table 2:** The influence of combined treatments of vermicompost and NPK fertilization on the mean values of number of leaves per branch, fresh weight of leaf (g), and yield of fresh leaves (g tree^−1^) of *Moringa oleifera* trees in the first and second seasons.

Treatments		Number of Leaves Branch^−1^		Fresh Weight of Leaf (g)		Yield of Fresh Leaves (g tree^−1^)	
Vermicompost (ton ha^−1^)	NPK (2 g L^−1^)	First Season	Second Season	First Season	Second Season	First Season	Second Season
Control	Control	6.36 ± 0.22 u	6.44 ± 0.04 u	3.09 ± 0.02 j	2.95 ± 0.12 r	33 ± 4 o	32 ± 1 t
	Mineral	6.46 ± 0.22 t	6.55 ± 0.04 t	3.80 ± 0.03 ij	3.63 ± 0.14 q	60 ± 7 no	59 ± 2 s
	Nano	7.07 ± 0.24 q	7.16 ± 0.05 q	5.04 ± 0.04 efghij	4.81 ± 0.19 m	130 ± 15 klm	128 ± 5 p
10	Control	6.66 ± 0.23 s	6.75 ± 0.04 s	4.01 ± 0.03 hij	3.83 ±0.15 p	64 ± 7 no	63 ± 3 s
	Mineral	7.67 ± 0.26 n	7.77 ± 0.05 n	4.32 ± 0.03 ghij	4.12 ± 0.16 o	97 ± 11 mn	95 ± 4 r
	Nano	8.08 ± 0.28 l	8.18 ± 0.05 l	5.96 ± 0.04 bcdefghi	5.69 ± 0.23 j	208 ± 24 j	205 ± 8 m
20	Control	6.87 ± 0.24 r	6.95 ± 0.04 r	4.73 ± 0.04 fghij	4.52 ± 0.18 n	112 ± 13 lm	111 ± 4 q
	Mineral	7.98 ± 0.27 m	8.08 ± 0.05 m	6.17 ± 0.05 abcdefgh	5.89 ± 0.23 i	234 ± 27 ij	230 ± 9 l
	Nano	8.58 ± 0.29 h	8.69 ± 0.05 h	6.99 ± 0.05 abcdef	6.68 ± 0.26 f	321 ± 37 fg	316 ± 12 i
30	Control	7.37 ± 0.25 p	7.47 ± 0.05 p	5.24 ± 0.04 efghij	5.01± 0.20 l	145 ± 17 kl	143 ± 6 o
	Mineral	8.18 ± 0.28 k	8.28 ± 0.05 k	6.38 ± 0.05 abcdefg	6.09 ± 0.24 h	263 ± 30 hi	259 ± 10 k
	Nano	8.99 ± 0.31 f	9.10 ± 0.06 f	7.51 ± 0.06 abcd	7.17 ± 0.28 d	414 ± 48 d	408 ± 16 f
40	Control	7.47 ± 0.26 o	7.57 ± 0.05 o	5.45 ± 0.04 cdefghi	5.20 ± 0.21 k	161 ± 19 k	159 ± 6 n
	Mineral	8.68 ± 0.30 g	8.79 ± 0.06 g	7.20 ± 0.05 abcde	6.87 ± 0.27 e	371 ± 43 e	366 ± 14 g
	Nano	9.89 ± 0.34 c	10.02 ± 0.06 c	8.02 ± 0.06 ab	7.66 ± 0.30 b	573 ± 66 b	565 ± 22 c
50	Control	8.38 ± 0.29 j	8.49 ± 0.05 j	6.68 ± 0.05 abcdef	6.38 ± 0.25 g	288 ± 33 gh	284 ± 11 j
	Mineral	9.19 ± 0.32 l	9.31 ± 0.06 e	7.71 ± 0.06 abc	7.36 ± 0.29 c	449 ± 52 d	442 ± 17 e
	Nano	10.00 ± 0.34 b	10.12 ± 0.06 b	8.23 ± 0.06 ab	7.85 ± 0.31 a	634 ± 73 a	625 ± 25 b
60	Control	8.48 ± 0.29 i	8.59 ± 0.05 i	7.10 ± 0.05 abcde	6.77 ± 0.27 ef	345 ± 40 ef	340 ± 13 h
	Mineral	9.29 ± 0.32 d	9.41 ± 0.06 d	7.82 ± 0.06 ab	7.46 ± 0.30 c	496 ± 57 c	488 ± 19 d
	Nano	10.10 ± 0.35 a	10.23 ± 0.06 a	8.33 ± 0.06 a	7.95 ± 0.31 a	674 ± 78 a	664 ± 26 a

At the 0.05 significance level, the means of columns separated by the same lowercase letter do not differ statistically. The data are mean values with standard error (*n* = 3).

**Table 3:** The influence of combined treatments of vermicompost and NPK fertilization on the mean values of yield of fresh leaves (kg ha^−1^), dry weight of leaf (g), and yield of dry leaves (g tree^−1^) of *Moringa oleifera* trees in the first and second seasons.

Treatments		Yield of Fresh Leaves (kg ha^−1^)		Dry Weight of Leaf (g)		Yield of Dry Leaves (g tree^−1^)	
Vermicompost (ton ha^−1^)	NPK (2 g L^−1^)	First Season	Second Season	First Season	Second Season	First Season	Second Season
Control	Control	328 ± 38 o	323 ± 13 t	0.93 ± 0.01 u	0.88 ± 0.03 r	9.8 ± 1.1o	9.7 ± 0.4 t
	Mineral	599 ± 69 no	590 ± 23 s	1.14 ± 0.01 t	1.09 ± 0.04 q	18.0 ± 2.1 no	17.7 ± 0.7 s
	Nano	1303 ± 150 klm	1285 ± 51 p	1.51 ± 0.01 p	1.44 ± 0.06 m	39.1 ± 4.5 klm	38.5 ± 1.5 p
10	Control	641 ± 74 no	632 ± 25 s	1.20 ± 0.01 s	1.15 ± 0.05 p	19.2 ± 2.2 no	18.9 ± 0.7 s
	Mineral	968 ± 112 mn	954 ± 38 r	1.30 ± 0.01 r	1.24 ± 0.05 o	29.0 ± 3.4 mn	28.6 ± 1.1 r
	Nano	2083 ± 240 j	2052 ± 81 m	1.79 ± 0.01 m	1.71 ± 0.07 j	62.5 ± 7.2 j	61.6 ± 2.4 m
20	Control	1124 ± 130 lm	1108 ± 44 lm	1.42 ± 0.01 q	1.35 ± 0.05 n	33.7 ± 3.9 lm	33.2 ± 1.3 q
	Mineral	2337 ± 270 ij	2303 ± 91 l	1.85 ± 0.01 l	1.77 ± 0.07 i	70.1 ± 8.1 ij	69.1 ± 2.7 l
	Nano	3206 ± 370 fg	3159 ± 125 i	2.10 ± 0.02 i	2.00 ± 0.08 f	96.2 ± 11.1 fg	94.8 ± 3.7 i
30	Control	1453 ± 168 kl	1432 ± 57 o	1.57 ± 0.01 o	1.50 ± 0.06 l	43.6 ± 5.0 kl	43.0 ± 1.7 o
	Mineral	2631 ± 304 hi	2592 ± 102 k	1.91 ± 0.01 k	1.83 ± 0.07 h	78.9 ± 9.1 hi	77.8 ± 3.1 k
	Nano	4137 ± 478 d	4077 ± 161 f	2.25 ± 0.02 f	2.15 ± 0.09 d	124.1 ± 14.3 d	122.3 ± 4.8 f
40	Control	1611 ± 186 k	1588 ± 63n	1.64 ± 0.01 n	1.56 ± 0.06 k	48.3 ± 5.6 k	47.6 ± 1.9 n
	Mineral	3710 ± 428 e	3656 ± 144g	2.16 ± 0.02 g	2.06 ± 0.08 e	111.3 ± 12.9 e	109.7 ± 4.3 g
	Nano	5731 ± 662 b	5648 ± 223c	2.41 ± 0.02 c	2.30 ± 0.09 b	171.9 ± 19.9 b	169.4 ± 6.7 c
50	Control	2881 ± 333 gh	2840 ± 112 j	2.01 ± 0.01 j	1.91 ± 0.08 g	86.4 ± 10.0 gh	85.2 ± 3.4 j
	Mineral	4486 ± 518 d	4421 ± 175 e	2.31 ± 0.02 e	2.21 ± 0.09 c	134.6 ± 15.5 d	132.6 ± 5.2 e
	Nano	6345 ± 733 a	6253 ± 247 b	2.47 ± 0.02 b	2.36 ± 0.09 a	190.3 ± 22.0 a	187.6 ± 7.4 b
60	Control	3453 ± 399 ef	3403 ± 134 h	2.13 ± 0.02 h	2.03 ± 0.08 ef	103.6 ± 12.0 ef	102.1 ± 4.0 h
	Mineral	4955 ± 572 c	4883 ± 193 d	2.34 ± 0.02 d	2.24 ± 0.09 c	148.7 ± 17.2 c	146.5 ± 5.8 d
	Nano	6739 ± 778 a	6641 ± 262 a	2.50 ± 0.02 a	2.39 ± 0.09 a	202.2 ± 23.3 a	199.2 ± 7.9 a

At the 0.05 significance level, the means of columns separated by the same lowercase letter do not differ statistically. The data are mean values with standard error (*n* = 3).

**Table 4:** The influence of combined treatments of vermicompost and NPK fertilization on the mean values of yield of dry leaves (kg ha^−1^), number of inflorescences per tree, fresh weight of inflorescences (g) of *Moringa oleifera* trees in the first and second seasons.

Treatments		Yield of Dry Leaves (kg ha^−1^)		Number of Inflorescences Per Tree		Fresh Weight of Inflorescences (g)	
Vermicompost (ton ha^−1^)	NPK (2 g L^−1^)	First Season	Second Season	First Season	Second Season	First Season	Second Season
Control	Control	98 ± 11 o	97 ± 4 t	6.3 ± 0.58 o	6.5 ± 0.13 t	2.07 ± 0.05 u	2.10 ± 0.04 u
	Mineral	180 ± 21 no	177 ± 7 s	9.3 ± 0.91 o	9.6 ± 0.20 s	2.59 ± 0.06 t	2.63 ± 0.05 t
	Nano	391 ± 45 klm	385 ± 15 p	16.5 ± 1.69 lmn	17.0 ± 0.37 p	3.63 ± 0.09 p	3.68 ± 0.07 p
10	Control	192 ± 22 no	189 ± 7 s	9.8 ± 0.96 o	10.1 ± 0.21 s	2.90 ± 0.07 s	2.94 ± 0.06 s
	Mineral	290 ± 34 mn	286 ± 11 r	13.8 ± 1.39 n	14.2 ± 0.31 r	3.11 ± 0.08 r	3.15 ± 0.06 r
	Nano	625 ± 72 j	616 ± 24 m	24.0 ± 2.51 k	24.8 ± 0.55 m	4.35 ± 0.11 m	4.41 ± 0.08 m
20	Control	337 ± 39 lm	332 ± 13 q	15.0 ± 1.53 mn	15.5 ± 0.34 q	3.42 ± 0.08 q	3.47 ± 0.07 q
	Mineral	701 ± 81 ij	691 ± 27 l	26.7 ± 2.80 jk	27.6 ± 0.62 l	4.56 ± 0.11 l	4.62 ± 0.09 l
	Nano	962 ± 111 fg	948 ± 37 i	34.4 ± 3.64 h	35.6 ± 0.80 i	5.08 ± 0.12 i	5.15 ± 0.10 i
30	Control	436 ± 50 kl	430 ± 17 o	18.2 ± 1.87 lm	18.7 ± 0.41 o	3.84 ± 0.09 o	3.89 ± 0.07 o
	Mineral	789 ± 91 hi	778 ± 31 k	29.9 ± 3.14 ij	30.8 ± 0.69 k	4.67 ± 0.11 k	4.73 ± 0.09 k
	Nano	1241 ± 143 d	1223 ± 48 f	43.4 ± 4.61 f	44.8 ± 1.02 f	5.70 ± 0.14 f	5.78 ± 0.11 f
40	Control	483 ± 56 k	476 ± 19 n	19.6 ± 2.03 l	20.2 ± 0.45 n	4.04 ± 0.10 n	4.10 ± 0.08 n
	Mineral	1113 ± 129 e	1097 ± 43 g	39.6 ± 4.20 g	40.9 ± 0.93 g	5.49 ± 0.13 g	5.57 ± 0.11 g
	Nano	1719 ± 199 b	1694 ± 67 c	61.7 ± 6.60 b	60.0 ± 1.37 c	6.32 ± 0.15 c	6.41 ± 0.12 c
50	Control	864 ± 100 gh	852 ± 34 j	32.0 ± 3.38 hi	34.0 ± 1.00 j	4.77± 0.12 j	4.83 ± 0.09 j
	Mineral	1346 ± 155 d	1326 ± 52 e	52.2 ± 5.57 d	52.1 ± 1.19 d	5.91 ± 0.14 e	5.99 ± 0.11 e
	Nano	1903 ± 220 a	1876 ± 74 a	70.6 ± 7.16 a	73.5 ± 1.69 a	6.53 ± 0.16 b	6.62 ± 0.13 b
60	Control	1036 ± 120 ef	1021± 40 ef	33.5 ± 1.00 h	39.1 ± 1.00 h	5.29 ± 0.13 h	5.36 ± 0.10 h
	Mineral	1487 ± 172 c	1465± 58 c	48.5 ± 5.17 e	49.4 ± 1.13 e	6.12 ± 0.15 d	6.20 ± 0.12 d
	Nano	2022 ± 233 a	1992± 79 a	57.3 ± 6.69 c	61.4 ± 1.74 b	6.95 ± 0.17 a	7.04 ± 0.13 a

At the 0.05 significance level, the means of columns separated by the same lowercase letter do not differ statistically. The data are mean values with standard error (*n* = 3).

**Table 5:** The influence of combined treatments of vermicompost and NPK fertilization on the mean values of yield of fresh inflorescences (g tree^−1^), yield of fresh inflorescences (kg ha^−1^), and dry weight of inflorescences (g) of *Moringa oleifera* trees in the first and second seasons.

Treatments		Yield of Fresh Inflorescences (g tree^−1^)		Yield of Fresh Inflorescences (kg ha^−1^)		Dry Weight of Inflorescences (g)	
Vermicompost (ton ha^−1^)	NPK (2 g L^−1^)	First Season	Second Season	First Season	Second Season	First Season	Second Season
Control	Control	13.1 ± 1.5 m	13.6 ± 0.3 q	131 ± 15 m	136 ± 3 q	0.30 ± 0.01 u	0.31 ± 0.01 u
	Mineral	24.2 ± 2.9 lm	25.2 ± 0.5 p	242 ± 29 lm	252 ± 5 p	0.46 ± 0.02 t	0.47 ± 0.01 t
	Nano	60.0 ± 7.4 ijk	62.6 ± 1.3 n	600 ± 74 ijk	626 ± 13 n	0.79 ± 0.03 q	0.80 ± 0.02 q
10	Control	28.4 ± 3.4 klm	29.6 ± 0.6 p	284 ± 34 klm	296 ± 6 p	0.55 ± 0.02 s	0.56 ± 0.02 s
	Mineral	42.9 ± 5.3 jklm	44.7 ± 0.9 o	429 ± 53 jklm	447 ± 9 o	0.62 ± 0.02 r	0.63 ± 0.02 r
	Nano	104.8 ± 13.2 gh	109.3 ± 2.4 l	1048 ± 132 gh	1093 ± 24 l	1.08 ± 0.03 m	1.09 ± 0.03 m
20	Control	51.5 ± 6.3 ijkl	53.6 ± 1.1 no	515 ± 63 ijkl	536 ± 11 no	0.86 ± 0.08 p	0.88 ± 0.06 p
	Mineral	122.2 ± 15.4 fg	127.5 ± 2.8 k	1222 ± 154 fg	1275 ± 28 k	1.15 ± 0.04 l	1.17 ± 0.03 l
	Nano	175.3 ± 22.3 e	182.9 ± 4.0 h	1753 ± 223 e	1829 ± 40 h	1.36 ± 0.04 i	1.38 ± 0.03 i
30	Control	69.8 ± 8.7 ij	72.8 ± 1.6 m	698 ± 87 ij	728 ± 16 m	0.90 ± 0.03 o	0.91 ± 0.02 o
	Mineral	139.6 ± 17.7 f	145.6 ± 3.2 j	1396 ± 177 f	1456 ± 32 j	1.20 ± 0.04 k	1.22 ± 0.03 k
	Nano	247.9 ± 31.7 d	258.8 ± 5.7 e	2479 ± 317 d	2588 ± 57 e	1.59 ± 0.05 f	1.62 ± 0.04 f
40	Control	79.4 ± 9.9 hi	82.8 ± 1.8 m	794 ± 99 hi	828 ± 18 m	0.97 ± 0.03 n	0.99 ± 0.02 n
	Mineral	218.1 ± 27.8 d	227.6 ± 5.0 f	2181 ± 278 d	2276 ± 50 f	1.51 ± 0.04 g	1.54 ± 0.04 g
	Nano	390.8 ± 50.2 b	400.3 ± 21.1 c	3908 ± 502 b	4003 ± 211 c	1.84 ± 0.05 c	1.86 ± 0.04 c
50	Control	152.9 ± 19.4 ef	164.3 ± 8.0 i	1529 ± 194 ef	1643 ± 80 i	1.25 ± 0.04 j	1.26 ± 0.03 j
	Mineral	308.7 ± 39.6 c	311.5 ± 6.8 d	3087 ± 396 c	3115 ± 68 d	1.67 ± 0.05 e	1.70 ± 0.04 e
	Nano	461.8 ± 56.8 a	485.9 ± 10.7 a	4618 ± 568 a	4859 ± 107 a	1.92 ±0.05 b	1.95 ± 0.04 b
60	Control	177.1 ± 9.6 e	209.7 ± 9.3 g	1771 ± 96 e	2097 ± 93 g	1.43 ± 0.04 h	1.46 ± 0.04 h
	Mineral	297.3 ± 38.1 c	306.3 ± 6.7 d	2973 ± 381 c	3063 ± 67 d	1.76 ± 0.05 d	1.78 ± 0.04 d
	Nano	398.3 ± 55.9a	432.1 ± 15.3 b	3983 ± 559 a	4321 ± 153 b	2.13 ± 0.06 a	2.16 ± 0.05 a

At the 0.05 significance level, the means of columns separated by the same lowercase letter do not differ statistically. The data are mean values with standard error (*n* = 3).

**Table 6:** The influence of combined treatments of vermicompost and NPK fertilization on the mean values of yield of dry inflorescences (g tree^−1^), yield of dry inflorescences (kg ha^−1^), and total chlorophyll (spad unit) of *Moringa oleifera* trees in the first and second seasons.

Treatments		Yield of Dry Inflorescences (g tree^−1^)		Yield of Dry Inflorescences (g ha^−1^)		Total Chlorophyll (spad unit)	
Vermicompost (ton ha^−1^)	NPK (2 g L^−1^)	First Season	Second Season	First Season	Second Season	First Season	Second Season
Control	Control	1.9 ± 0.3 n	2.0 ± 0.1 r	19 ± 3 n	20 ±1 r	28.4 ± 0.4 u	28.5 ± 0.1 u
	Mineral	4.3 ± 0.6 mn	4.5 ± 0.1 qr	43 ± 6 mn	45 ± 1 qr	30.5 ± 0.5 t	30.6 ± 0.1 t
	Nano	13.0 ±1.7 klm	13.7 ± 0.3 o	130 ± 17 klm	137 ± 3 o	33.4 ± 0.5 p	33.5 ± 0.2 p
10	Control	5.4 ± 0.7 mn	5.7 ± 0.2 q	54 ± 7 mn	57 ± 2 q	31.5 ± 0.5 s	31.6 ± 0.2 s
	Mineral	8.6 ± 1.1 lmn	9.0 ± 0.2 p	86 ± 11 lmn	90 ± 2 p	32.6 ± 0.5 r	32.7 ± 0.2 r
	Nano	25.9 ± 3.4 ij	27.1 ± 0.7 l	259 ± 34 ij	271 ± 7 l	35.0 ± 0.5 m	35.1 ± 0.2 m
20	Control	13.0 ± 2.4 klm	13.6 ± 0.8 o	130 ± 24 klm	136 ± 8 o	32.9 ± 0.5 q	33.0 ± 0.2 q
	Mineral	30.8 ± 4.1 hi	32.3 ± 0.8 k	308 ± 41 hi	323 ± 8 k	35.2 ± 0.5 l	35.3 ± 0.2 l
	Nano	46.8 ± 6.2 f	49.0 ± 1.2 h	468 ± 62 f	490 ± 12 h	36.8 ± 0.6 i	36.9 ± 0.2 i
30	Control	16.3 ± 2.2 kl	17.1 ± 0.4 n	163 ± 22 kl	171 ± 4 n	34.7 ± 0.5 o	34.8 ± 0.2 o
	Mineral	36.0 ± 4.8 gh	37.6 ± 0.9 j	360 ± 48 gh	376 ± 9 j	36.3 ± 0.6 k	36.4 ± 0.2 k
	Nano	69.3 ± 9.1 d	72.5 ± 1.7 e	693 ± 91 d	725 ± 17 e	38.8 ± 0.6 f	38.9 ± 0.2 f
40	Control	19.1 ± 2.5 jk	19.9 ± 0.5 m	191 ± 25 jk	199 ± 5 m	34.9 ± 0.5 n	35.0 ± 0.2 n
	Mineral	60.1 ± 7.9 e	62.8 ± 1.5 f	601 ± 79 e	628 ± 15 f	38.3 ± 0.6 g	38.4 ± 0.2 g
	Nano	113.6 ± 15.0 b	111.9 ± 2.6 c	1136 ± 150 b	1119 ± 26 c	39.7 ± 0.6 c	39.9 ± 0.2 c
50	Control	39.9 ± 5.3 fg	43.0 ± 2.3 i	399 ± 53 fg	430 ± 23 i	36.6 ± 0.6 j	36.7 ± 0.2 j
	Mineral	87.5 ± 11.6 c	88.4 ± 2.1 d	875 ± 116 c	884 ± 21d	39.0 ± 0.6 e	39.1 ± 0.2 e
	Nano	135.8 ± 17.2 a	143.2 ± 3.3 a	1358 ± 172 a	1432 ± 33 a	40.0 ± 0.6 b	40.1 ± 0.2 b
60	Control	48.0 ± 2.8 f	57.2 ± 3.0 g	480 ± 28 f	572 ± 30 g	37.8 ± 0.6 h	38.0 ± 0.2 h
	Mineral	85.3 ± 11.2 c	88.1 ± 2.0 d	853 ± 112 c	881 ± 20 d	39.2 ± 0.6 d	39.3 ± 0.2 d
	Nano	122.2 ± 6.5 b	132.8 ± 4.9 b	1222 ± 65 b	1328 ± 49 b	40.3 ± 0.6 a	40.4 ± 0.2 a

At the 0.05 significance level, the means in columns separated by the same lowercase letters are not statistically different. The data are mean values with standard error (*n* = 3).

**Table 7:** The influence of combined treatments of vermicompost and NPK fertilization on the mean values of leaf soluble protein (mg g^−1^) and vitamin C (mg g^−1^ dry weight) of *Moringa oleifera* trees in the first and second seasons.

Treatments		Leaf Soluble Protein (mg g^−1^)		Vitamin C (mg g^−1^ Dry Weight)	
Vermicompost (ton ha^−1^)	NPK (2 g L^−1^)	First Season	Second Season	First Season	Second Season
Control	Control	17.5 ± 0.4 u	17.7 ± 0.4 u	27.7 ± 2.2 p	28.9 ± 0.4 u
	Mineral	19.5 ± 0.5 t	19.8 ± 0.5 t	29.0 ± 2.3 o	30.3 ± 0.4 t
	Nano	22.5 ± 0.6 p	22.8 ± 0.6 p	35.2 ± 2.7 l	36.8 ± 0.5 p
10	Control	20.6 ± 0.5 s	20.8 ± 0.5 s	30.0 ± 2.3 n	31.4 ± 0.5 s
	Mineral	21.6 ± 0.5 r	21.9 ± 0.5 r	33.1 ± 2.6 m	34.6 ± 0.5 r
	Nano	23.6 ± 0.6 m	23.9 ± 0.6 m	39.7 ± 3.1 j	41.5 ± 0.6 m
20	Control	22.0 ± 0.5 q	22.3 ± 0.5 q	33.5 ± 2.6 m	35.0 ± 0.5 q
	Mineral	23.8 ± 0.6 l	24.1 ± 0.6 l	40.8 ± 3.2 i	42.7 ± 0.6 l
	Nano	24.8 ± 0.6 i	25.1 ± 0.6 i	44.0 ± 3.4 fg	46.0 ± 0.7 i
30	Control	22.9 ± 0.6 o	23.2 ± 0.6 o	38.2 ± 3.0 k	40.0 ± 0.6 o
	Mineral	24.2 ± 0.6 k	24.5 ± 0.6 k	42.5 ± 3.3 h	44.4 ± 0.7 k
	Nano	26.5 ± 0.7 f	26.9 ± 0.7 f	45.5 ± 3.5 e	47.5 ± 0.7 f
40	Control	23.3 ± 0.6 n	23.6 ± 0.6 n	38.7 ± 3.0 k	40.5 ± 0.6 n
	Mineral	26.0 ± 0.6 g	26.4 ± 0.6 g	45.3 ± 3.5 e	47.3 ± 0.7 g
	Nano	27.8 ± 0.7 c	28.1 ± 0.7 c	48.3 ± 3.8 c	50.5 ± 0.7 c
50	Control	24.5 ± 0.6 j	24.8 ± 0.6 j	43.1 ± 3.4 gh	45.1 ± 0.7 j
	Mineral	26.9 ± 0.7 e	27.3 ± 0.7 e	47.2 ± 3.7 d	49.4 ± 0.7 e
	Nano	28.1 ± 0.7 b	28.4 ± 0.7 b	51.5 ± 4.0 b	53.8 ± 0.8 b
60	Control	25.2 ± 0.6 h	25.5 ± 0.6 h	44.7 ± 3.5 ef	46.7 ± 0.7 h
	Mineral	27.6 ± 0.7 d	27.9 ± 0.7 d	47.6 ± 3.7 cd	49.8 ± 0.7 d
	Nano	28.9 ± 0.7 a	29.3 ± 0.7 a	53.7 ± 4.2 a	56.1 ± 0.8 a

At the 0.05 significance level, the means in columns separated by the same lowercase letters are not statistically different. The data are mean values with standard error (*n* = 3).

**Table 8:** The influence of combined treatments of vermicompost and NPK fertilization on the mean values of total phenolic (mg Gallic g^−1^ dry herb), flavonoid content (mg Rutin g^−1^ dry herb), and antioxidant activity (IC_50_^b^ of *Moringa oleifera* trees in the first and second seasons).

Treatments		Total Phenolic (mg Gallic 1 g^−1^ dry herb)		Flavonoids (mg Rutin g^−1^ dry herb)		Antioxidant activity (IC_50_^b^)	
Vermicompost (ton ha^−1^)	NPK (2 g L^−1^)	First Season	Second Season	First Season	Second Season	First Season	Second Season
Control	Control	47.12 ± 1.67 a	48.23 ± 1.67 a	28.01 ± 0.99 a	28.67 ± 0.99 a	30.70 ± 1.09 r	31.42 ± 1.09 r
	Mineral	45.30 ± 1.60 b	46.36 ± 1.60 b	27.61 ± 0.98 b	28.26 ± 0.98 b	33.03 ± 1.17 q	33.81 ± 1.17 q
	Nano	41.02 ± 1.45 e	41.99 ± 1.45 f	23.74 ± 0.84 d	24.30 ± 0.84 d	36.98 ± 1.31 n	37.85 ± 1.31 n
10	Control	44.90 ± 1.59 b	45.95 ± 1.59 c	27.32 ± 0.97 b	27.96 ± 0.97 b	34.04 ± 1.20 p	34.84 ± 1.20 p
	Mineral	43.21 ± 1.53 c	44.23 ± 1.53 d	25.13 ± 0.89 c	25.72 ± 0.89 c	33.64 ± 1.19 p	34.43 ± 1.19 p
	Nano	37.75 ± 1.33 h	38.63 ± 1.33 i	21.16 ± 0.75 g	21.66 ± 0.75 g	38.70 ± 1.37 l	39.61 ± 1.37 l
20	Control	41.83 ± 1.48 d	42.81 ± 1.48 e	25.43 ± 0.90 c	26.03 ± 0.90 c	35.06 ± 1.24 o	35.88 ± 1.24 o
	Mineral	35.76 ± 1.26 i	36.60 ± 1.26 j	18.87 ± 0.67 h	19.32 ± 0.67 h	39.51 ± 1.40 k	40.44 ± 1.40 k
	Nano	32.78 ± 1.16 l	33.55 ± 1.16 m	17.38 ± 0.61 j	17.79 ± 0.61 j	44.59 ± 1.58 h	45.64 ± 1.58 h
30	Control	39.73 ± 1.40 f	40.67 ± 1.40 g	22.95 ± 0.81 e	23.49 ± 0.81 e	36.68 ± 1.30 n	37.54 ± 1.30 n
	Mineral	34.87 ± 1.23 j	35.69 ± 1.23 k	18.77 ± 0. 66 h	19.22 ± 0.66 h	40.73 ± 1.44 j	41.69 ± 1.44 j
	Nano	31.29 ± 1.11 n	32.03 ± 1.11 p	14.40 ± 0.51 m	14.74 ± 0.51 m	47.62 ± 1.68 f	48.74 ± 1.68 f
40	Control	39.14 ± 1.38 g	40.06 ± 1.38 h	22.25 ± 0.79 f	22.77 ± 0.79 f	37.59 ± 1.33 m	38.47 ± 1.33 m
	Mineral	31.69 ± 1.12 n	32.43 ± 1.12 o	15.79 ± 0.56 l	16.17 ± 0.56 l	46.91 ± 1.66 g	48.01 ± 1.66 g
	Nano	29.60 ± 1.05 p	30.30 ± 1.05 r	12.81 ± 0.45 p	13.12 ± 0.45 p	51.98 ± 1.84 c	53.20 ± 1.84 c
50	Control	33.97 ± 1.20 k	34.77 ± 1.20 l	18.18 ± 0.64 i	18.61 ± 0.64 i	43.57 ± 1.54 i	44.59 ± 1.54 i
	Mineral	30.40 ± 1.07 o	31.11 ± 1.07 q	13.81 ± 0.49 n	14.13 ± 0.49 n	49.24 ± 1.74 e	50.40 ± 1.74 e
	Nano	26.32 ± 0.93 q	26.94 ± 0.93 s	12.22 ± 0.43 q	12.51 ± 0.43 q	56.74 ± 2.01 b	58.07 ± 2.01 b
60	Control	32.28 ± 1.14 m	33.04 ± 1.14 n	16.69 ± 0.59 k	17.08 ± 0.59 k	46.61 ± 1.65 g	47.70 ± 1.65 g
	Mineral	29.90 ± 1.06 p	30.60 ± 1.06 r	13.41 ± 0.47 o	13.73 ± 0.47 o	50.46 ± 1.78 d	51.64 ± 1.78 d
	Nano	24.83 ± 0.88 r	25.42 ± 0.88 t	12.02 ± 0.42 q	12.30 ± 0.42 q	58.77 ± 2.08 a	60.15 ± 2.08 a

At the 0.05 significance level, the means of columns separated by the same lowercase letter do not differ statistically. The data are mean values with standard error (*n* = 3).

**Table 9:** The influence of combined treatments of vermicompost and NPK fertilization on the mean values of phosphorus content (% P_2_O_5_) and potassium content (% K_2_O) of *Moringa oleifera* trees in the first and second seasons. The mean values of phosphorus content (% P_2_O_5_) and potassium content (% K_2_O) of *Moringa oleifera* leaves as affected with organic fertilization and mineral fertilization combination treatments in both seasons of the study.

Treatments		Phosphorus (% P_2_O_5_)		Potassium (% K_2_O)	
Vermicompost (ton ha^−1^)	NPK (2 g L^−1^)	First Season	Second Season	First Season	Second Season
Control	Control	0.189 ± 0.017 q	0.207 ± 0.003 u	2.08 ± 0.18 n	2.27 ± 0.03 t
	Mineral	0.217 ± 0.018 o	0.238 ± 0.003 t	2.25 ± 0.20 lm	2.47 ± 0.04 r
	Nano	0.232 ± 0.021 m	0.254 ± 0.004 p	2.40 ± 0.21 jk	2.62 ± 0.04 o
10	Control	0.198 ± 0.019 p	0.217 ± 0.004 s	2.21 ± 0.20 m	2.42 ± 0.04 s
	Mineral	0.222 ± 0.020 n	0.243 ± 0.004 r	2.28 ± 0.20 lm	2.50 ± 0.04 q
	Nano	0.247 ± 0.022 j	0.271 ± 0.004 m	2.63 ± 0.23 h	2.88 ± 0.04 l
20	Control	0.228 ± 0.020 m	0.250 ± 0.004 q	2.32 ± 0.21 kl	2.54 ± 0.04 p
	Mineral	0.250 ± 0.022 ij	0.274 ± 0.004 l	2.66 ± 0.24 gh	2.91 ± 0.04 k
	Nano	0.258 ± 0.023 fg	0.283 ± 0.004 i	2.89 ± 0.26 e	3.16 ± 0.05 h
30	Control	0.237 ± 0.021 l	0.259 ± 0.004 o	2.47 ± 0.22 ij	2.71 ± 0.04 n
	Mineral	0.254 ± 0.023 hi	0.278 ± 0.004 k	2.72 ± 0.24 g	2.98 ± 0.04 j
	Nano	0.267 ± 0.024 cd	0.292 ± 0.004 f	3.41 ± 0.30 b	3.74 ± 0.06 e
40	Control	0.242 ± 0.022 k	0.266 ± 0.004 n	2.53 ± 0.22 i	2.77 ± 0.04 m
	Mineral	0.264 ± 0.023 de	0.289 ± 0.004 g	3.33 ± 0.30 c	3.65 ± 0.05 f
	Nano	0.273 ± 0.024 b	0.299 ± 0.004 c	3.58 ± 0.32 a	3.92 ± 0.06 c
50	Control	0.256 ± 0.023 gh	0.280 ± 0.004 j	2.80 ± 0.25 f	3.07 ± 0.05 i
	Mineral	0.269 ± 0.024 bc	0.295 ± 0.004 e	3.48 ± 0.31 b	3.81 ± 0.06 d
	Nano	0.278 ± 0.025 a	0.305 ± 0.005 b	3.60 ± 0.32 a	3.95 ± 0.06 b
60	Control	0.262 ± 0.023 ef	0.287 ± 0.004 h	3.12 ± 0.28 d	3.42 ± 0.05 g
	Mineral	0.271 ± 0.024 bc	0.297 ± 0.004 d	3.49 ± 0.31 b	3.82 ± 0.06 d
	Nano	0.282 ± 0.025 a	0.309 ± 0.005 a	3.63 ± 0.32 a	3.98 ± 0.06 a

At the 0.05 significance level, the means of columns separated by the same lowercase letter do not differ statistically. The data are mean values with standard error (*n* = 3).

**Table 10:** All different combination treatments of vermicompost and NPK fertilization used.

Treatments	Main-Plot	Sub-Plot
T1	0 ton ha^−1^ vermicompost	0 g L^−1^ NPK
T2		2 g L^−1^ NPK
T3		2 g L^−1^ Nano-NPK
T4	10 ton ha^−1^ vermicompost	0 g L^−1^ NPK
T5		2 g L^−1^ NPK
T6		2 g L^−1^ Nano-NPK
T7	20 ton ha^−1^ vermicompost	0 g L^−1^ NPK
T8		2 g L^−1^ NPK
T9		2 g L^−1^ Nano-NPK
T10	30 ton ha^−1^ vermicompost	0 g L^−1^ NPK
T11		2 g L^−1^ NPK
T12		2 g L^−1^ Nano-NPK
T13	40 ton ha^−1^ vermicompost	0 g L^−1^ NPK
T14		2 g L^−1^ NPK
T15		2 g L^−1^ Nano-NPK
T16	50 ton ha^−1^ vermicompost	0 g L^−1^ NPK
T17		2 g L^−1^ NPK
T18		2 g L^−1^ Nano-NPK
T19	60 ton ha^−1^ vermicompost	0 g L^−1^ NPK
T20		2 g L^−1^ NPK
T21		2 g L^−1^ Nano-NPK

**Table 11:** The physical and chemical properties of the used vermicompost.

Vermicompost Property		
Organic matter	%	41.57
C	%	17.02
N	%	1.82
Mn	%	0.03
B	mg g^−1^	0.054
Ca	mg g^−1^	19.57
Cu	mg g^−1^	0.25
Fe	mg g^−1^	1.27
Mg	mg g^−1^	6.01
Na	mg g^−1^	1.48
P2O5	mg g^−1^	4.61
K	mg g^−1^	1.93
Ec	ds m^−1^	1.78
pH		7.2

**Table 12:** The physical and chemical properties of the experimental soil.

Soil Property		
O.M.		0.75
Sand	%	65.3
Silt	%	15.8
Clay	%	18.9
Texture class	Sandy clay loam	
pH		8.51
Ec	ds m^−1^	1.72
N	%	0.032
P_2_O_4_	mg g^−1^	0.004
K^+^	mg g^−1^	0.287
Fe	mg g^−1^	0.0038
Zn	mg g^−1^	0.0014
Mn	mg g^−1^	0.0035
Cu	mg g^−1^	0.00059
B	mg g^−1^	0.0003

## Data Availability

The data presented in this study are available within the article.
